# Supplementary material for: Music Interventions for Anxiety in Pregnant Women: A Systematic Review and Meta-Analysis of Randomized Controlled Trials
Source: J Clin Med. 2019 Nov 6;8(11):1884. doi: 10.3390/jcm8111884 (PMC6912569; doi:10.3390/jcm8111884)
Supplement: Supplementary file 1 [file jcm-08-01884-s001.pdf]

Supplementary Materials

Table S1. PRISMA checklist.

Section/Topic.	#	Checklist Item	Reported on Page #
		Title	
	1	Identify the report as a systematic review, meta-analysis, or both.	1
		Abstract	
Structured summary	2	Provide a structured summary including, as applicable: background; objectives; data sources; study eligibility criteria, participants, and interventions; study appraisal and synthesis methods; results; limitations; conclusions and implications of key findings; systematic review registration number.	1
		Introduction	
Rationale	3	Describe the rationale for the review in the context of what is already known.	1–2
Objectives	4	Provide an explicit statement of questions being addressed with reference to participants, interventions, comparisons, outcomes, and study design (PICOS).	2
		Methods	
Protocol and registration	5	Indicate if a review protocol exists, if and where it can be accessed (e.g., Web address), and, if available, provide registration information including registration number.	NA
Eligibility criteria	6	Specify study characteristics (e.g., PICOS, length of follow-up) and report characteristics (e.g., years considered, language, publication status) used as criteria for eligibility, giving rationale.	2
Information sources	7	Describe all information sources (e.g., databases with dates of coverage, contact with study authors to identify additional studies) in the search and date last searched.	2
Search	8	Present full electronic search strategy for at least one database, including any limits used, such that it could be repeated.	Table S2
Study selection	9	State the process for selecting studies (i.e., screening, eligibility, included in systematic review, and, if applicable, included in the meta-analysis).	2, Figure 1
Data collection process	10	Describe method of data extraction from reports (e.g., piloted forms, independently, in duplicate) and any processes for obtaining and confirming data from investigators.	2
Data items	11	List and define all variables for which data were sought (e.g., PICOS, funding sources) and any assumptions and simplifications made.	2, 5
Risk of bias in individual studies	12	Describe methods used for assessing risk of bias of individual studies (including specification of whether this was done at the study or outcome level), and how this information is to be used in any data synthesis.	5
Summary measures	13	State the principal summary measures (e.g., risk ratio, difference in means).	5
Synthesis of results	14	Describe the methods of handling data and combining results of studies, if done, including measures of consistency (e.g., I^2) for each meta-analysis.	5

Risk of bias across studies	15	Specify any assessment of risk of bias that may affect the cumulative evidence (e.g., publication bias, selective reporting within studies).	5
Additional analyses	16	Describe methods of additional analyses (e.g., sensitivity or subgroup analyses, meta-regression), if done, indicating which were pre-specified.	5
		Results	
Study selection	17	Give numbers of studies screened, assessed for eligibility, and included in the review, with reasons for exclusions at each stage, ideally with a flow diagram.	5–6, Figure 1
Study characteristics	18	For each study, present characteristics for which data were extracted (e.g., study size, PICOS, follow-up period) and provide the citations.	3–6, Table 1
Risk of bias within studies	19	Present data on risk of bias of each study and, if available, any outcome level assessment (see item 12).	7, Table S3
Results of individual studies	20	For all outcomes considered (benefits or harms), present, for each study: (a) simple summary data for each intervention group (b) effect estimates and confidence intervals, ideally with a forest plot.	7, Figure 2
Synthesis of results	21	Present results of each meta-analysis done, including confidence intervals and measures of consistency.	7
Risk of bias across studies	22	Present results of any assessment of risk of bias across studies (see Item 15).	7, Figure S3
Additional analysis	23	Give results of additional analyses, if done (e.g., sensitivity or subgroup analyses, meta-regression [see Item 16]).	7–8, Figure 3, Figure 4, Figure S1, Figure S2
		Discussion	
Summary of evidence	24	Summarize the main findings including the strength of evidence for each main outcome; consider their relevance to key groups (e.g., healthcare providers, users, and policy makers).	8–9
Limitations	25	Discuss limitations at study and outcome level (e.g., risk of bias), and at review-level (e.g., incomplete retrieval of identified research, reporting bias).	10
Conclusions	26	Provide a general interpretation of the results in the context of other evidence, and implications for future research.	10
		Funding	
Funding	27	Describe sources of funding for the systematic review and other support (e.g., supply of data); role of funders for the systematic review.	10

Table S2. Search strategy.

**Search strategy in Pubmed**	
**Search.**	**Query**
#120	Search ((((((((((((((((((((((pregnancy) OR Pregnancies) OR Gestation) OR Fertilization) OR Postnatal) OR Postpartum) OR Antenatal) OR perinatal) OR antepartum) OR peripartum) OR Pregnant Women) OR Labor, Obstetric) OR delivery) OR labor) OR Parturition) OR childbirth) OR birth*) OR puerperium) OR doing month)) OR (Cesarean Section OR abdominal deliver* OR C-Section OR C Section OR Caesarean Section*))) AND (((((((music therapy) OR music) OR lullaby) OR rhythm[Title/Abstract]) OR melody[Title/Abstract]) OR Jazz[Title/Abstract]) OR song[Title/Abstract]))) AND (((((((((((((((Anxiety) OR Anxiety) OR Hypervigilance) OR Nervousness) OR Stress, Psychological) OR Mental Suffering) OR emotional stress) OR life stress) OR Expressed Emotion) OR Irritable Mood) OR self-rating anxiety scale) OR State-Trait Anxiety Inventory) OR STAI[Title/Abstract]) OR Test Anxiety Scale) OR Test Anxiety Questionnaire) Sort by: [pubsolr12]
#121	Search (((Cesarean Section OR abdominal deliver* OR C-Section OR C Section OR Caesarean Section*)) AND (((((((music therapy) OR music) OR lullaby) OR rhythm[Title/Abstract]) OR melody[Title/Abstract]) OR Jazz[Title/Abstract]) OR song[Title/Abstract])) AND (((((((((((((((Anxiety) OR Anxiety) OR Hypervigilance) OR Nervousness) OR Stress, Psychological) OR Mental Suffering) OR emotional stress) OR life stress) OR Expressed Emotion) OR Irritable Mood) OR self-rating anxiety scale) OR State-Trait Anxiety Inventory) OR STAI[Title/Abstract]) OR Test Anxiety Scale) OR Test Anxiety Questionnaire)
#117	Search Cesarean Section OR abdominal deliver* OR C-Section OR C Section OR Caesarean Section*
#112	Search ((((((((((((((((((((pregnancy) OR Pregnancies) OR Gestation) OR Fertilization) OR Postnatal) OR Postpartum) OR Antenatal) OR perinatal) OR antepartum) OR peripartum) OR Pregnant Women) OR Labor, Obstetric) OR delivery) OR labor) OR Parturition) OR childbirth) OR birth*) OR puerperium) OR doing month)) AND (((((((music therapy) OR music) OR lullaby) OR rhythm[Title/Abstract]) OR melody[Title/Abstract]) OR Jazz[Title/Abstract]) OR song[Title/Abstract])) AND (((((((((((((((Anxiety) OR Anxiety) OR Hypervigilance) OR Nervousness) OR Stress, Psychological) OR Mental Suffering) OR emotional stress) OR life stress) OR Expressed Emotion) OR Irritable Mood) OR self-rating anxiety scale) OR State-Trait Anxiety Inventory) OR STAI[Title/Abstract]) OR Test Anxiety Scale) OR Test Anxiety Questionnaire) Sort by: [pubsolr12]
#110	Search ((((((((((((((Anxiety) OR Anxiety) OR Hypervigilance) OR Nervousness) OR Stress, Psychological) OR Mental Suffering) OR emotional stress) OR life stress) OR Expressed Emotion) OR Irritable Mood) OR self-rating anxiety scale) OR State-Trait Anxiety Inventory) OR STAI[Title/Abstract]) OR Test Anxiety Scale) OR Test Anxiety Questionnaire
#109	Search Test Anxiety Questionnaire
#108	Search Test Anxiety Scale
#105	Search STAI[Title/Abstract]
#104	Search State-Trait Anxiety Inventory
#102	Search self-rating anxiety scale
#101	Search Irritable Mood
#99	Search Expressed Emotion
#96	Search life stress
#95	Search emotional stress

#94	Search Mental Suffering
#93	Search Stress, Psychological
#90	Search Nervousness
#89	Search Hypervigilance
#88	Search Anxiety
#87	Search Anxiety Sort by: [pubsolr12]
#84	Search ((((((music therapy) OR music) OR lullaby) OR rhythm[Title/Abstract]) OR melody[Title/Abstract]) OR Jazz[Title/Abstract]) OR song[Title/Abstract]
#83	Search song[Title/Abstract]
#82	Search Jazz[Title/Abstract]
#81	Search melody[Title/Abstract]
#80	Search rhythm[Title/Abstract]
#79	Search lullaby
#78	Search music
#77	Search music therapy
#76	Search (((((((((((((((((pregnancy) OR Pregnancies) OR Gestation) OR Fertilization) OR Postnatal) OR Postpartum) OR Antenatal) OR perinatal) OR antepartum) OR peripartum) OR Pregnant Women) OR Labor, Obstetric) OR delivery) OR labor) OR Parturition) OR childbirth) OR birth*) OR puerperium) OR doing month
#75	Search doing month
#74	Search puerperium
#73	Search birth* Sort by: [pubsolr12]
#71	Search childbirth Sort by: [relevance]
#72	Search Parturition Sort by: [pubsolr12]
#70	Search labor Sort by: [pubsolr12]
#66	Search delivery Sort by: [pubsolr12]
#64	Search Labor, Obstetric Sort by: [pubsolr12]
#61	Search Pregnant Women Sort by: [pubsolr12]
#57	Search peripartum Sort by: [pubsolr12]
#56	Search antepartum Sort by: [pubsolr12]
#55	Search perinatal Sort by: [pubsolr12]
#53	Search Antenatal Sort by: [pubsolr12]
#50	Search Postpartum Sort by: [pubsolr12]
#49	Search Postnatal Sort by: [pubsolr12]
#46	Search Fertilization Sort by: [pubsolr12]
#43	Search Gestation Sort by: [pubsolr12]

#42	Search Pregnancies Sort by: [pubsolr12]
#39	Search pregnancy Sort by: [pubsolr12]
**Search strategy in Embase**	
#28	#13 AND #19 AND #27
#27	#20 OR #21 OR #22 OR #23 OR #24 OR #25 OR #26
#26	**anxiety'**:ti,ab,kw OR **'hypervigilance'**:ti,ab,kw OR **'nervousness'**:ti,ab,kw OR **'stress, psychological'**:ti,ab,kw OR **'emotional stress'**:ti,ab,kw OR **'life stress'**:ti,ab,kw OR **'mental suffering'**:ti,ab,kw OR **'emotions'**:ti,ab,kw OR **'irritable mood'**:ti,ab,kw OR **'expressed emotion'**:ti,ab,kw OR **'test anxiety scale'**:ti,ab,kw OR **'state-trait anxiety inventory'**:ti,ab,kw OR **'stai'**:ti,ab,kw OR **'mental health'**:ti,ab,kw
#25	**'state trait anxiety inventory'**/exp
#24	**'anxiety assessment'**/exp
#23	**'life stress'**/exp
#22	**'mental stress'**/exp
#21	**'anxiety disorder'**/exp
#20	**'anxiety'**/exp
#19	#14 OR #15 OR #16 OR #17 OR #18
#18	((**'music'** NEAR/2 **'therapy'**):ti,ab,kw) OR **'music'**:ti,ab,kw OR **'lullaby'**:ti,ab,kw OR **'rhythm'**:ti,ab,kw OR **'melody'**:ti,ab,kw OR **'singing'**:ti,ab,kw OR **'song'**:ti,ab,kw OR **'jazz'**:ti,ab,kw
#17	**'melody'**/exp
#16	**'singing'**/exp
#15	**'music'**/exp
#14	**'music therapy'**/exp
#13	#1 OR #2 OR #3 OR #4 OR #5 OR #6 OR #7 OR #8 OR #9 OR #10 OR #11 OR #12
#12	**'postnatal'**:ti,ab,kw OR **'antenatal'**:ti,ab,kw OR **'perinatal'**:ti,ab,kw OR **'antepartum'**:ti,ab,kw OR **'peripartum'**:ti,ab,kw OR **'postpartum'**:ti,ab,kw OR **'puerperium'**:ti,ab,kw OR **'pregnancy'**:ti,ab,kw OR **'gestation'**:ti,ab,kw OR **'fertilization'**:ti,ab,kw OR **'birth'**:ti,ab,kw OR **'pregnant'**:ti,ab,kw OR **'delivery'**:ti,ab,kw OR **'childbirth'**:ti,ab,kw OR **'labou'**:ti,ab,kw OR **'doin month'**:ti,ab,kw OR **'expectant mother*'**:ti,ab,kw OR **'cesarean section'**:ti,ab,kw OR **'c-section'**:ti,ab,kw OR **'caesarean section*'**:ti,ab,kw OR **'labor stage'**:ti,ab,kw
#11	**'cesarean section'**/exp
#10	**'postnatal care'**/exp
#9	**'expectant mother'**/exp
#8	**'perinatal period'**/exp
#7	**'childbirth'**/exp
#6	**'fertilization'**/exp
#5	**'obstetric delivery'**/exp
#4	**'labor stage'**/exp
#3	**'labor'**/exp
#2	**'pregnant woman'**/exp
#1	**'pregnancy'**/exp
**Search strategy in Cochrane Library**	

	Query	
#1	MeSH descriptor: [Pregnancy] explode all trees	
#2	MeSH descriptor: [Fertilization] explode all trees	
#3	MeSH descriptor: [Pregnant Women] explode all trees	
#4	MeSH descriptor: [Labor, Obstetric] explode all trees	
#5	MeSH descriptor: [Cesarean Section] explode all trees	
#6	pregnancy or Pregnancies or Gestation or Fertilization or Postnatal or Postpartum or Antenatal or perinatal or antepartum or peripartum or Pregnant Women or Labor, Obstetric or delivery or labor or Parturition or childbirth or birth* or puerperium or doing month or Cesarean Section or abdominal deliver* or C-Section or C Section or Caesarean Section* :ti,ab,kw (Word variations have been searched)	
#7	#1 or #2 or #3 or #4 or #5 or #6	
#8	MeSH descriptor: [Music Therapy] explode all trees	
#9	MeSH descriptor: [Music] explode all trees	
#10	music therapy or music or lullaby or rhythm or melody or Jazz or song:ti,ab,kw (Word variations have been searched)	
#11	#8 or #9 or #10	
#12	MeSH descriptor: [Anxiety] explode all trees	
#13	MeSH descriptor: [Stress, Physiological] explode all trees	
#14	MeSH descriptor: [Test Anxiety Scale] explode all trees	
#15	Anxiety or Hypervigilance or Nervousness or Stress, Psychological or Mental Suffering or emotional stress or life stress or Expressed Emotion or Irritable Mood or self-rating anxiety scale or State-Trait Anxiety Inventory or STAI or Test Anxiety Scale or Test Anxiety Questionnaire:ti,ab,kw (Word variations have been searched)	
#16	#12 or #13 or #14 or #15	
#17	#7 and #11 and #16	
**Search Strategy in CINAHL**		
S22	S12 AND S16 AND S21	Search modes - Find all my search terms
S21	S17 OR S18 OR S19 OR S20	Search modes - Find all my search terms
S20	TI (Anxiety OR Hypervigilance OR Nervousness OR Stress, Psychological OR Mental Suffering OR emotional stress OR life stress OR Expressed Emotion OR Irritable Mood OR self-rating anxiety scale OR State-Trait Anxiety Inventory OR STAI OR Test Anxiety Scale OR Test Anxiety Questionnaire) OR AB (Anxiety OR Hypervigilance OR Nervousness OR Stress, Psychological OR Mental Suffering OR emotional stress OR life stress OR Expressed Emotion OR Irritable Mood OR self-rating anxiety scale OR State-Trait	Search modes - Find all my search terms
S19	(MH "Self-Rating Anxiety Scale")	Search modes - Find all my search terms
S18	(MH "Stress, Physiological")	Search modes - Find all my search terms
S17	(MH "Anxiety") OR (MH "Anxiety Disorders")	Search modes - Find all my search terms
S16	S13 OR S14 OR S15	Search modes - Boolean/Phrase

S15	TI (music therapy OR music OR lullaby OR rhythm OR melody OR Jazz OR song) OR AB (music therapy OR music OR lullaby OR rhythm OR melody OR Jazz OR song)OR AB music therapy OR music OR lullaby OR rhythm OR melody OR Jazz OR song) OR AB (music therapy OR music OR lullaby OR rhythm OR melody OR Jazz OR song	Search modes - Find all my search terms
S14	(MH "Music") OR (MH "Singing")	Search modes - Find all my search terms
S13	(MH "Music Therapy")	Search modes - Find all my search terms
S12	S1 OR S2 OR S3 OR S4 OR S5 OR S6 OR S7 OR S8 OR S9 OR S10 OR S11 OR Cesarean Section OR abdominal deliver* OR C-Section OR C Section OR Caesarean Section*	Search modes - Boolean/Phrase
S11	AB pregnancy OR Pregnancies OR Gestation OR Fertilization OR Postnatal OR Postpartum OR Antenatal OR perinatal OR antepartum OR peripartum OR Pregnant Women OR Labor, Obstetric OR delivery OR labor OR Parturition OR childbirth OR birth* OR puerperium OR doing month	Search modes - Find all my search terms
S10	TI pregnancy OR Pregnancies OR Gestation OR Fertilization OR Postnatal OR Postpartum OR Antenatal OR perinatal OR antepartum OR peripartum OR Pregnant Women OR Labor, Obstetric OR delivery OR labor OR Parturition OR childbirth OR birth* OR puerperium OR doing month	Search modes - Find all my search terms
S9	(MH "Childbirth")	Search modes - Find all my search terms
S8	(MH "Puerperium")	Search modes - Find all my search terms
S7	(MH "Postpartum (Omaha)")	Search modes - Find all my search terms
S6	(MH "Perinatal Care")	Search modes - Find all my search terms
S5	(MH "Prenatal Care")	Search modes - Find all my search terms
S4	(MH "Labor") OR "labor" OR (MH "Labor Stage, Third") OR (MH "Labor Stage, Second") OR (MH "Labor Stage, First")	Search modes - Find all my search terms
S3	(MH "Expectant Mothers")	Search modes - Find all my search terms
S2	(MM "Fertilization")	Search modes - Find all my search terms
S1	(MH "Pregnancy+")	Search modes - Find all my search terms

Search Strategy in PsycINFO

**Search ID#**	**Search Terms**	**Search Options**
S4	S1 AND S2 AND S3	Search modes - Find all my search terms
S3	TI/AB (Anxiety OR Hypervigilance OR Nervousness OR Stress, Psychological OR Mental Suffering OR emotional stress OR life stress OR Expressed Emotion OR Irritable Mood OR self-rating anxiety scale OR State-Trait Anxiety Inventory OR STAI OR Test Anxiety Scale OR Test Anxiety Questionnaire)	Search modes - Find all my search terms

S2	TI/AB(music therapy OR music OR lullaby OR rhythm OR melody OR Jazz OR song)	Search modes - Find all my search terms
S1	TI/AB(pregnancy OR Pregnancies OR Gestation OR Fertilization OR Postnatal OR Postpartum OR Antenatal OR perinatal OR antepartum OR peripartum OR Pregnant Women OR Labor, Obstetric OR delivery OR labor OR Parturition OR childbirth OR birth* OR puerperium OR doing month)	Search modes - Find all my search terms
**Search Strategy in Airiti Library**		
	生產 OR 懷孕 OR 孕婦 OR 妊娠 OR 產婦 OR 產程 OR 產後 OR 坐月子 OR 安胎	
	音樂 OR 唱歌 OR 旋律 OR 爵士	
**Search Strategy in NDLTD**		
	生產 + 懷孕 + 孕婦 + 妊娠 + 產婦 + 產程 + 產後 + 坐月子 + 安胎	
	音樂 + 唱歌 + 旋律 + 爵士	

Table S3. Revised Cochrane risk-of-bias tool (RoB 2.0) for quality assessment of included RCTs.

Domain1.	Risk of bias arising from the randomization process					
Domain2	Risk of bias due to deviations from the intended interventions					
Domain3	Risk of bias due to missing outcome data					
Domain4	Risk of bias in measurement of the outcome					
Domain5	Risk of bias in selection of the reported result					
Overall risk of bias	Low risk of bias	The study is judged to be at low risk of bias for all domains for this result.				
	Some concerns	The study is judged to raise some concerns in at least one domain for this result, but not to be at high risk of bias for any domain.				
	High risk of bias	The study is judged to be at high risk of bias in at least one domain for this result. Or the study is judged to have some concerns for multiple domains in a way that substantially lowers confidence in the result.				
	**Domain 1**	**Domain 2**	**Domain 3**	**Domain 4**	**Domain 5**	**Overall risk**
Chang et al. 2008	Some concern	Some concern	Low	High	Some concern	High
Yang et al. 2009	Low	Some concern	Low	High	Some concern	High
Kafali et al. 2011	Some concern	Some concern	Low	High	Some concern	High
Guerrero et al. 2012	Some concern	Some concern	Low	High	Low	High
Wu et al. 2012	Some concern	Some concern	Low	High	Some concern	High
Cao et al. 2016	Low	Some concern	Low	High	Some concern	High

Liu et al. 2016	Some concern	Some concern	Low	High	Some concern	High
Toker and Kömürcü 2017	Low	Some concern	Low	High	Some concern	High
Nwebube et al. 2017	Some concern	Some concern	High	High	Low	High
Garcia-Gonzalez et al. 2018	Low	Some concern	Low	High	Some concern	High
Teckenberg-Jansson et al. 2019	Some concern	Some concern	Low	High	Some concern	High

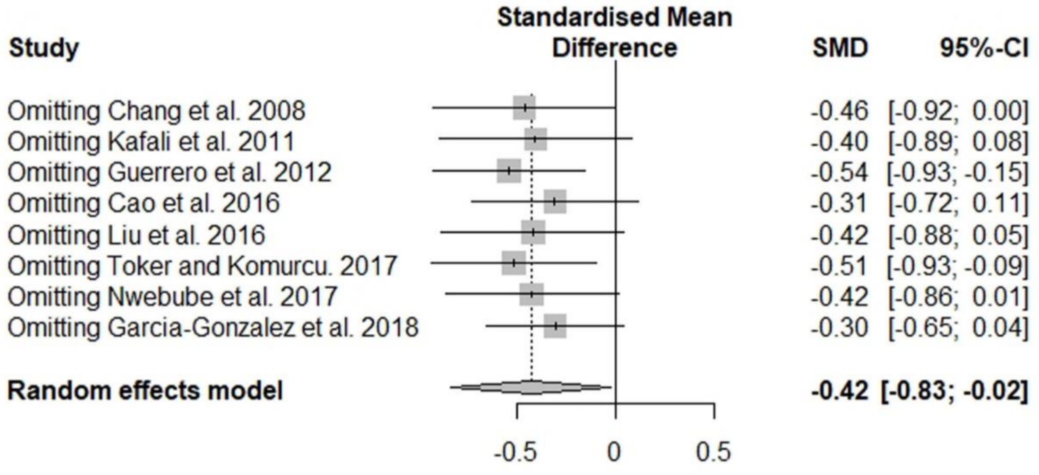

Figure S1. Sensitivity analyses of music interventions on anxiety in pregnant women by omitting each study. SMD, standardized mean difference; CI, confidence interval.

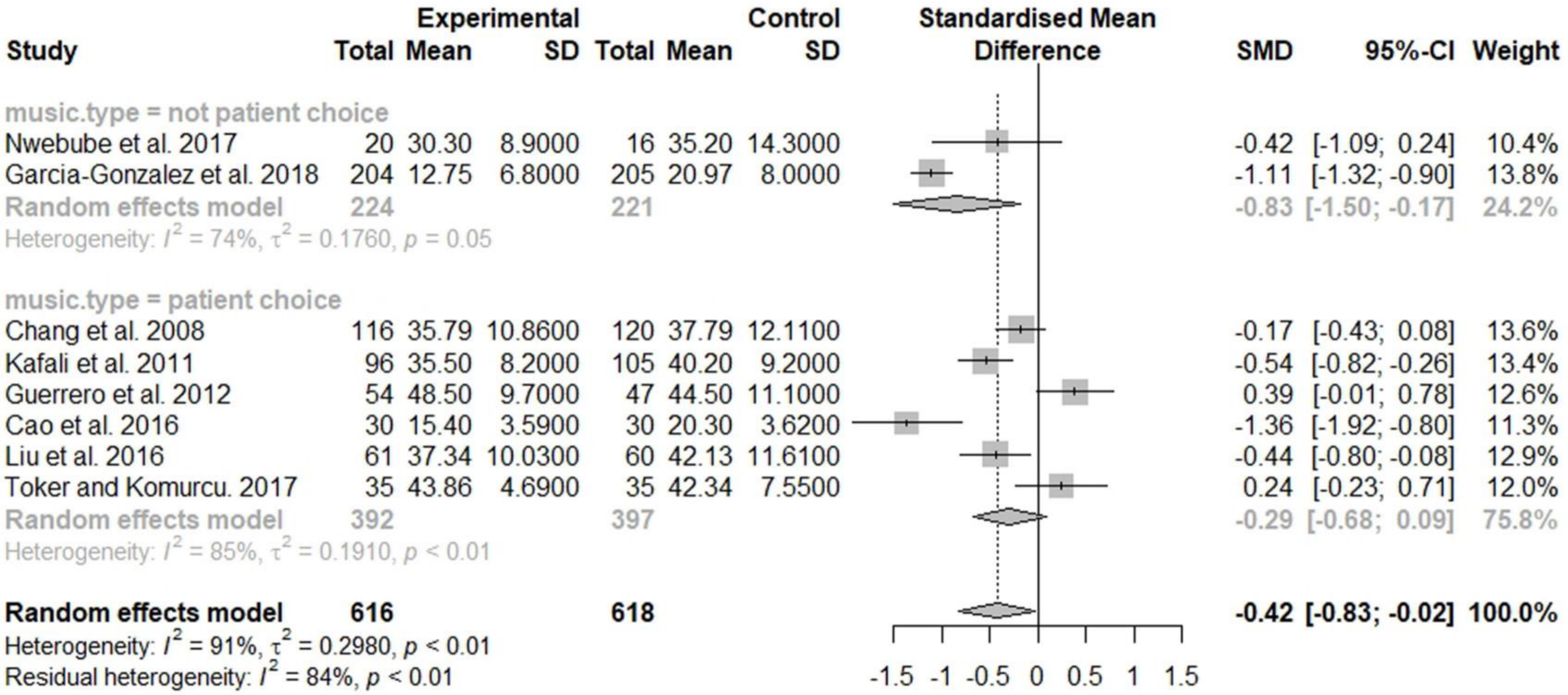

Figure S2. Forest plot of pooled anxiety scores after the intervention, comparing the music group and the control group (subgroup analysis by the music type). SMD, standardized mean difference; CI, confidence interval.

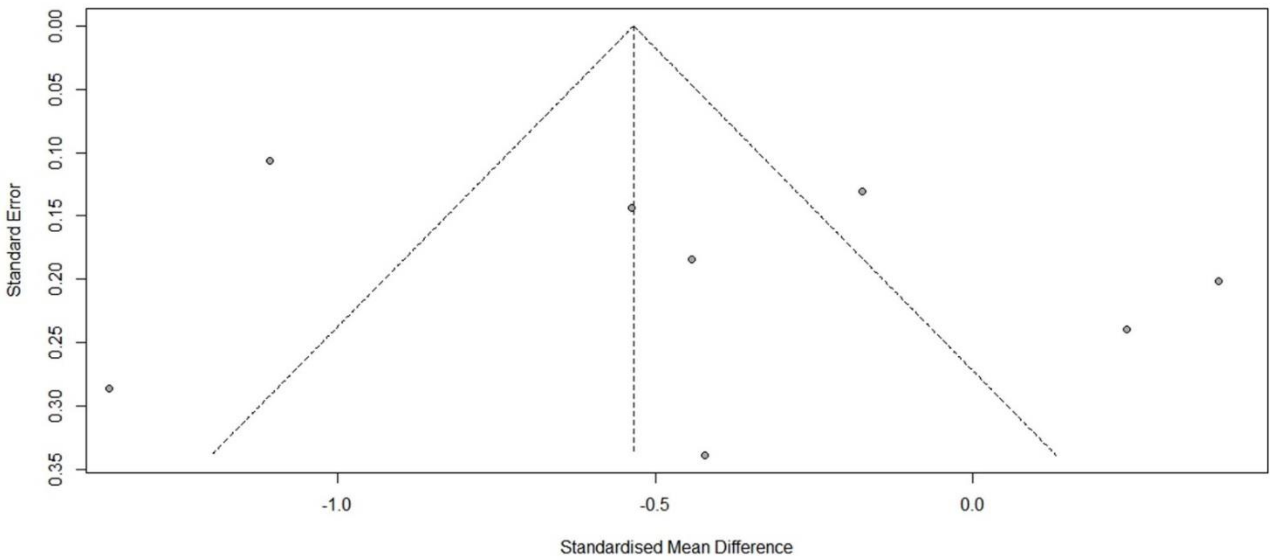

Figure S3. The Funnel plot of the standard error by standardized mean difference of the studies included in the meta-analysis. Egger's test, slope=-1.04, p = 0.38.
